# AL and ATTR cardiac amyloid are different: native T1 mapping and ECV detect different biology

**DOI:** 10.1186/1532-429X-16-S1-P341

**Published:** 2014-01-16

**Authors:** Marianna Fontana, Sanjay M Banypersad, Thomas A Treibel, Viviana Maestrini, Daniel Sado, Steven K White, Heerajnarain Bulluck, Anna S Herrey, Philip N Hawkins, James Moon

**Affiliations:** 1 Heart Hospital Imaging Center, Heart Hospital, University College London, London, UK; 2 National Amyloidosis Center, Royal Free Hospital, University College London, London, UK

## Background

Cardiac involvement drives prognosis in amyloidosis. ECV directly quantitates interstitial expansion and hence myocardial amyloid burden. Native T1 is also elevated in amyloid but reflects both cell and interstitial changes. The combination gives insight into amyloid burden and the myocyte response. Previously we have shown the utility of both techniques in AL amyloidosis. Here, we explore the differences between the two main types of amyloid, AL and ATTR types.

## Methods

3 groups were studied: ATTR amyloid patients (n = 102; age 72 ± 10); transthyretin mutations carriers (n = 8; age 47 ± 6); AL amyloid patients (n = 81; age 62 ± 10). These were compared with 52 healthy volunteers and 43 patients with hypertrophic cardiomyopathy (HCM). All underwent T1 mapping and ECV measurement. ATTR patients and mutation carriers also underwent DPD scintigraphy.

## Results

**Figure 1:**
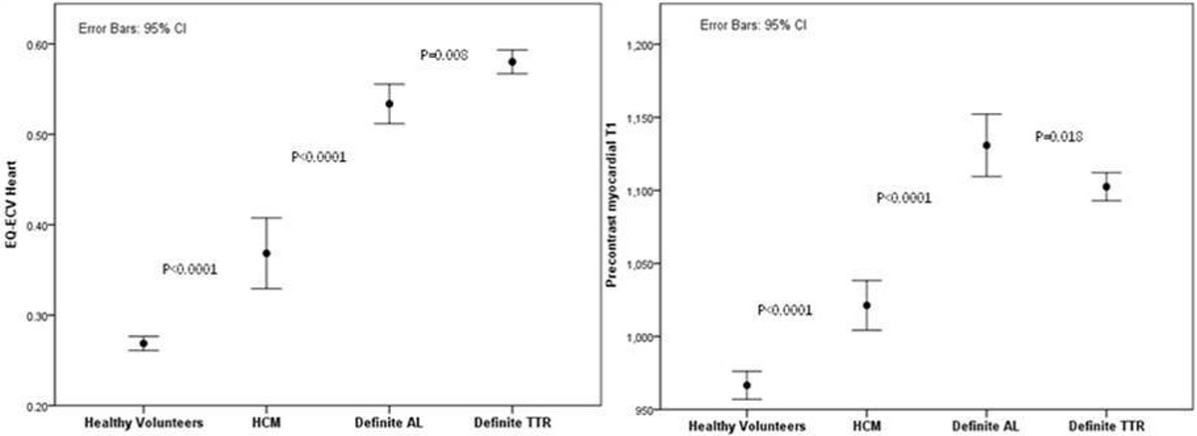
**ECV (left panel) and native myocardial T1 (right panel) in healthy volunteers, HCM, definite AL and definite ATTR amyloidosis**.

ECV was massively elevated in ATTR patients compared to HCM and healthy volunteers (0.58 ± 0.06 ms vs 0.37 ± 0.12 ms vs 0.27 ± 0.03 ms, both p > 0.0001). In established cardiac ATTR amyloidosis, ECV elevation was higher than AL amyloidosis (AL 0.53 ± 0.07 ms, p = 0.008) (Figure 1). Conversely, T1 was lower in TTR than AL amyloidosis (Figure 1). Diagnostic performance of ECV was similar for AL and TTR (vs HCM: AL AUC 0.824 (0.745-0.902); TTR AUC 0.805 (95%CI 0.748-0.862); both P < 0.0001). ECV tracked cardiac amyloid burden as determined by DPD scintigraphy. ECV was not elevated in mutation carriers (0.27 ± 0.02 ms) but was in isolated DPD grade 1 (n = 8, 0.37 ± 0.09 ms, p = 0.001). Correlations between ECV and other parameters showed specific differences between AL and ATTR (Table 1).

**Table 1:** 

	ATTR patients (R)	AL patients (R)
LV structure by MRI		

LA areai, cm2/m2	0.695*	0.435*

LV systolic function by CMR	0.410*	0.258†

LVEF, %	-0.523*	0.504*

SVi, ml/m2	-0.428*	-0.428*

LV diastolic function by echo		

E/E'	0.512*	0.365*

E-deceleration time, ms	-0.240 †	-0.252†

6 minutes walking test	-0.357*	Ns

Biomarkers		

NT-proBNP, pmol/L	0.789*	0.670*

Troponin T, pmol/L	0.681*	0.531*

ECG		

PR, ms	0.472*	ns

QRS, ms	0.281 *	ns

ECG limb lead mean voltage	-0.263 †	-0.424 *

* P < 0.01 level; †P < 0.05;

## Conclusions

ECV detects cardiac ATTR amyloid with similar diagnostic performance and disease tracking to T1. The ECV is higher in TTR - i.e. there is proportionately more amyloid in TTR than AL hearts. However the native T1 is lower. The discordance of ECV and T1 in AL and ATTR cardiac amyloidosis highlights a possible difference in the myocyte response, giving unique insight into the pathophysiology of cardiac amyloidosis.

## Funding

Dr Fontana Is funded by the British Heart Foundation. A proportion of the scans have been funded by GlaxoSmithKline.

